# Linear plasmids in *Klebsiella* and other *Enterobacteriaceae*

**DOI:** 10.1099/mgen.0.000807

**Published:** 2022-04-13

**Authors:** Jane Hawkey, Hugh Cottingham, Alex Tokolyi, Ryan R. Wick, Louise M. Judd, Louise Cerdeira, Doroti de Oliveira Garcia, Kelly L. Wyres, Kathryn E. Holt

**Affiliations:** ^1^​ Department of Infectious Diseases, Central Clinical School, Monash University, Melbourne, Victoria 3004, Australia; ^2^​ Department of Human Genetics, Wellcome Sanger Institute, Hinxton, UK; ^3^​ Liverpool School of Tropical Medicine, Liverpool L3 5QA, UK; ^4^​ Regional Laboratory Center, Adolfo Lutz Institute, Marilia, Brazil; ^5^​ Department of Infection Biology, London School of Hygiene and Tropical Medicine, London WC1E 7HT, UK

**Keywords:** *Enterobacteriaceae*, *Klebsiella*, linear plasmids, toxin–antitoxin

## Abstract

Linear plasmids are extrachromosomal DNA elements that have been found in a small number of bacterial species. To date, the only linear plasmids described in the family *Enterobacteriaceae* belong to *Salmonella *, first found in *Salmonella enterica* Typhi. Here, we describe a collection of 12 isolates of the *Klebsiella pneumoniae* species complex in which we identified linear plasmids. Screening of assembly graphs assembled from public read sets identified linear plasmid structures in a further 13 *K *. *pneumoniae* species complex genomes. We used these 25 linear plasmid sequences to query all bacterial genome assemblies in the National Center for Biotechnology Information database, and discovered an additional 61 linear plasmid sequences in a variety of *Enterobacteriaceae* species. Gene content analysis divided these plasmids into five distinct phylogroups, with very few genes shared across more than two phylogroups. The majority of linear plasmid-encoded genes are of unknown function; however, each phylogroup carried its own unique toxin–antitoxin system and genes with homology to those encoding the ParAB plasmid stability system. Passage *in vitro* of the 12 linear plasmid-carrying *Klebsiella* isolates in our collection (which include representatives of all five phylogroups) indicated that these linear plasmids can be stably maintained, and our data suggest they can transmit between *K. pneumoniae* strains (including members of globally disseminated multidrug-resistant clones) and also between diverse *Enterobacteriaceae* species. The linear plasmid sequences, and representative isolates harbouring them, are made available as a resource to facilitate future studies on the evolution and function of these novel plasmids.

## Significance as a BioResource to the community

This study provides, to the best of our knowledge, the first report of linear plasmids identified within the *Klebsiella pneumoniae* species complex and the first report in *Enterobacteriaceae* besides *Salmonella *. We present the first comparative analysis of linear plasmid sequences in *Enterobacteriaceae *; however, whilst this family is highly clinically significant, the functional and/or evolutionary importance of these plasmids is not yet clear. To facilitate future studies to address these questions, we have publicly deposited: (i) the collection of linear plasmid sequence data; (ii) isolates representative of each of the distinct linear plasmid phylogroups.

## Data Summary

1. Whole-genome sequence reads from *Klebsiella pneumoniae* isolates sequenced in this study have been deposited in the National Center for Biotechnology Information (NCBI) SRA (sequence read archive) under the accession numbers listed in Table S1 (available with the online version of this article).
2. Representative annotated sequences of one linear plasmid per phylogroup have been deposited in FigShare (https://doi.org/10.26180/16729126).
3. A copy of all linear plasmid sequences that we assembled from publicly available genome sequence reads are available in FigShare (https://doi.org/10.26180/16531365). Read accessions for these are given in Table S1.
4. Eleven representative *K. pneumoniae* isolates harbouring linear plasmids described in this study have been deposited with the National Collection of Type Cultures (NCTC) and are available for purchase under the NCTC accession numbers listed in Table S1. *K. pneumoniae* 1194/11 (representative of phylogroup B) has been deposited in the Microorganisms Collection Center, Adolfo Lutz Institute, São Paulo, Brazil. To request strain 1194/11 (IAL 3063, SISGEN ABBF09B), contact: Culture Collection Laboratory, Microorganisms Collection Center, Adolfo Lutz Institute, São Paulo State Department of Health, Room 1020, 10th Floor, Avenida Dr Arnaldo, 351, São Paulo 01246 000, Brazil (phone number +55-11-3068 2884; e-mail colecaoial@ial.sp.gov.br).
5. Alignments of terminal inverted repeat sequences for each phylogroup can be found in Data S1, available on FigShare (https://doi.org/10.26180/16531371).

## Introduction

Plasmids are extrachromosomal DNA elements that are frequently found in bacterial cells. The vast majority of plasmid molecules exist in a circular conformation; however, linear plasmids have been found in several bacterial species, with the first description in *Streptomyces* in 1979 [1], and later in *Borrelia* [2] (where they are universally present) and *Rhodococcus* [3]. A study of clinical *Enterococcus faecium* isolates recently reported the existence of a 143 kbp linear plasmid carrying a *N*-acetylgalactosamine (GalNAc) utilization operon that could be transferred between strains via conjugation [4]. Linear plasmids appear to be exceedingly rare within *Enterobacteriaceae *, with the first, pBSSB1 (27 kbp), described in 2007 from *Salmonella enterica* Typhi isolated in Indonesia [5]. Prior to this discovery, the only other linear replicons described within *Enterobacteriaceae* were those derived from bacteriophage, including pKO2 in *Klebsiella oxytoca* [6], N15 in *Escherichia coli* [7] and PY54 in *Yersinia enterocolitica* [8]. These bacteriophage-derived linear replicons are distinct from the true linear plasmids described in *Salmonella *, *Enterococcus *, *Streptomyces* and *Borrelia *, as they still possess bacteriophage-specific genes including those for the lysis pathways [6].

For replicons that are linear, there is a requirement to stabilize the terminal ends to ensure stability and appropriate replication, which in eukaryotes is achieved through the use of telomeres. In contrast, bacterial linear plasmids can either (i) create hairpin loops, as in *Borrelia* [9] and *Enterococcus* [4], or (ii) bind telomere-associated proteins to each end of the molecule with the assistance of terminal inverted repeats (TIRs), as in *Streptomyces* [10]. The *Salmonella* linear plasmid pBSSB1 was found to carry 1230 bp TIRs with covalently bound proteins on the end, similar to *Streptomyces *; however, these had no homology to any previously identified TIRs [5].

The *Salmonella enterica* Typhi linear plasmid pBSSB1 encodes two flagellar genes, an *fljA*-like gene and *fljB^z66^* [5]. *fljB^z66^* encodes the phase II z66 flagellin antigen, whilst the *fljA­*-like gene is thought to encode the repressor of the chromosomally encoded phase I flagellin antigen, allowing for phase II z66 antigen presentation [5]. Few other genes from the 27 kbp plasmid pBSSB1 have been characterized, and no replication system has been described. Linear plasmids homologous to pBSSB1 have since been described in other *Salmonella* serovars, at a prevalence of ~0.3 %, the majority of which carried the z66 flagellin genes [11].

In this study, we report the discovery of multiple diverse linear plasmids in genomes belonging to the *Klebsiella pneumoniae* species complex (* K. pneumoniae* and six closely related taxa) within the *Enterobacteriaceae *. We demonstrate the linearity of these replicons using long-read and short-read sequencing, show they are reliably maintained within their natural host isolates during 10 rounds of laboratory passage, and identify homologues in the genomes of several other *Enterobacteriaceae* species. Clustering on the basis of gene content, we identify five major phylogroups of *K. pneumoniae* linear plasmids and describe their sequence characteristics in terms of size, G+C content, TIR sequence and TIR length.

## Methods

### Identifying linear plasmids in *K. pneumoniae* species complex genomes

**Fig. 1.**
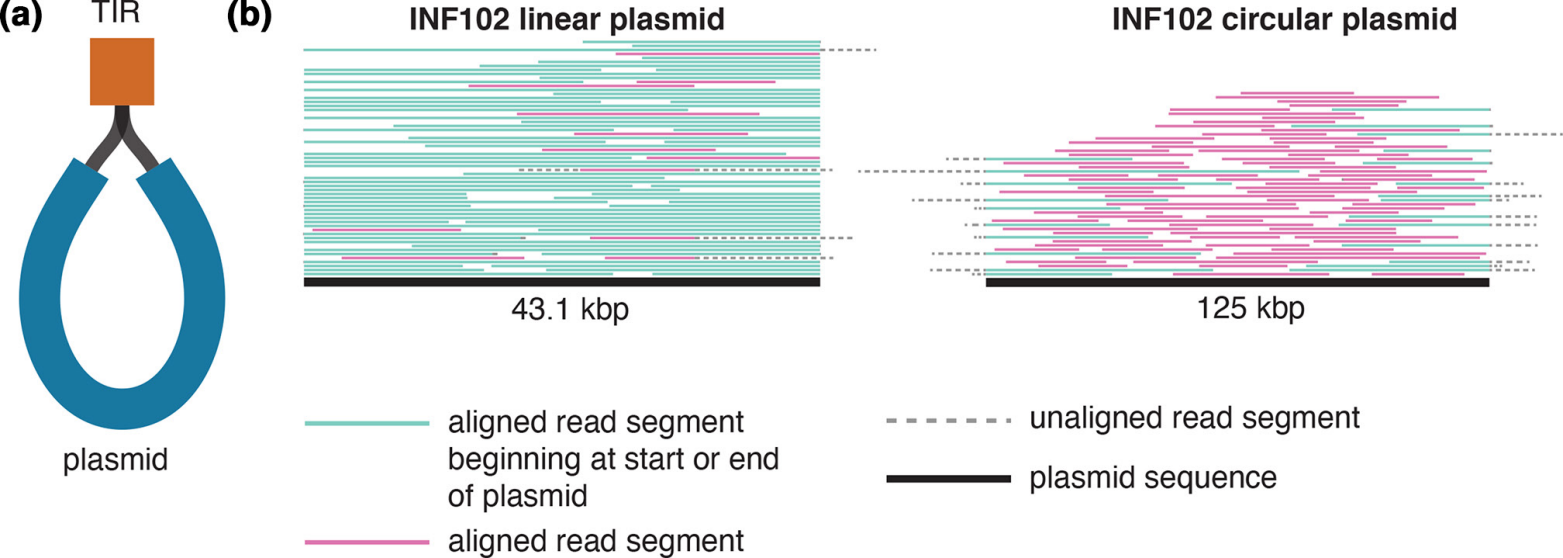
Using read sequence data to determine linearity of plasmid sequences. (a) Short-read assembly graph structure of a linear plasmid. The plasmid consists of two contigs, the main plasmid sequence (blue, labelled plasmid), connected to a second, shorter contig that is the TIR at both ends (orange, labelled TIR). (**b) **Long reads aligned to the linear plasmid sequence and a representative circular plasmid sequence from INF102, with the total number of alignments shown capped at 100 to improve visualization. The plasmid sequence is the thick black line at the bottom, and reads aligning to the plasmid are shown in green if the alignment starts at the beginning or end of the plasmid sequence, or pink if the alignment starts elsewhere. Segments coloured dotted grey indicate regions of the read that do not align. Alignments to the linear plasmid have very few reads that soft-clip off the ends of the plasmid sequence, indicating linearity. Conversely, alignments to a circular plasmid have many reads soft-clipping over the edges of the plasmid sequence, indicating that this replicon is circular.

We screened for linear plasmids in the assembly graphs of 1119 genomes of the *K. pneumoniae* species complex, including 460 from our own collection of human clinical and carriage isolates [12–15] and 667 publicly available read sets (see Table 1). Paired-end Illumina reads for each genome were assembled using Unicycler v0.4.7 [16], using default parameters. The first assembly graph produced by Unicycler (001_best_spades_graph.gfa) was searched for the signature two-contig structure of a linear plasmid (a connected component of the graph consisting of one contig connected at both ends to the same end of another contig, see Fig. 1a) using a custom Python script (available at doi 10.26180/16531374). We subsequently used these linear plasmid sequences as queries for a nucleotide blast search of the 1119 genome assemblies, to recover instances where the linear plasmid sequence was present but had not fully assembled into the characteristic two-contig graph structure. This resulted in a total of 25 linear plasmid sequences, these have been deposited in FigShare (doi 10.26180/16531365).

**Table 1.** Number of *Klebsiella* species complex genomes in which a linear plasmid structure was detected from assembly graphs, across multiple different studies from a variety of geographical regions and sampling types

Dataset	No. of genomes	No. of linear plasmids	Country of origin	Sampling type
In-house collection (KASPAH) [12, 13]	452	11 (2.4 %)	Australia	Humans (infections and faecal carriage)
Bueno 2013 [14]	8	1 (12.5 %)	Brazil	Humans, agricultural animals, urban waterways
Stoesser 2013 [43]	69	2 (2.9 %)	UK	Humans (bloodstream infections)
Smit 2018 [44]	90	3 (3.3 %)	Cambodia	Humans (neonatal care unit)
Davis 2015 [45]	61	1 (1.6 %)	UK	Humans (urinary tract infections) and retail meat
Henson 2017 [46]	185	5 (2.7 %)	Kenya	Humans (bloodstream infections)
Moradigaravand 2017 [47]	250	2 (0.8 %)	UK and Ireland	Humans (bloodstream infections)
Total assembly graphs screened	1115	25 (2.2 %)	–	–

### Identifying homologues in other species

To detect homologous linear plasmids in other bacterial species, we performed a nucleotide blast search of National Center for Biotechnology Information (NCBI) sequences (May 10th 2021), using as queries each of the linear plasmid sequences identified in *Klebsiella *, as well as the pBSSB1 sequence (accession no. NC_011422). Hits with ≥90 % identity and ≥60 % coverage of a query sequence were considered as putative linear plasmid sequences (*n*=61). Metadata for each linear plasmid sequence and its host bacterium was pulled from the GenBank record for the corresponding whole-genome sequence. To confirm the taxonomy and multilocus sequence types of the bacterial hosts of these putative linear plasmids, the chromosome sequence for each genome was uploaded to Pathogenwatch (https://pathogen.watch). For strain WP3-W18-ESBL-02 (in which plasmid 3, accession no. AP021975.1, was a hit to linear plasmid query pINF007 plasmid 3), Pathogenwatch was unable to detect a species; however, the Genome Taxonomy Database (using GTDB-Tk [17] with database release 202, https://gtdb.ecogenomic.org) assigned it as a novel *Kluyvera* species, *Kluyvera ascorbata_B*. Table S1 lists the species given by the submitter in GenBank, in addition to species detected by Pathogenwatch or GTDB, for all genomes.

### Plasmid stability analysis

For the 12 bacterial isolates in our collection with linear plasmids, we tested the stability of these plasmids during 10 passages in broth culture. Isolates from frozen glycerol stocks were streaked onto cation adjusted Mueller Hinton (CAMH) agar plates and incubated for 20 h at 37 °C. A single colony from each plate was streaked onto a fresh CAMH plate and inoculated into 3 ml CAMH broth, and both were incubated for 20 h at 37 °C. From the broth culture, a glycerol stock and bacterial pellet, day 1 (D1) samples were prepared. This process was repeated nine additional times to yield day 2–10 (D2–D10) samples.

Long-read sequencing (Oxford Nanopore Technologies; ONT) was performed as previously described [18]. Briefly, genomic DNA was prepared from the D1 and D10 bacterial pellets using GenFind v3 reagents (Beckman Coulter). A long-read sequencing library was prepared using the ligation library kit (LSK-109; ONT) with native barcoding expansion pack (EXP-NBD104 and NBD114; ONT). The library was run on a R9.4.1 MinION flow cell for 48 h yielding 2.75 Gbp of reads. Reads were basecalled with Guppy v3.3.3 using the dna_r9.4.1_450bps_hac (high-accuracy) basecalling model.

To determine presence/absence and copy number of all plasmids in each genome, reads were mapped to their respective reference genome assemblies (listed in Table S1) using minimap2 v2.17 [19]. Mean read depth across each replicon in the assembly was calculated using the read alignments, and the copy number for each plasmid was determined by dividing mean read depth across a plasmid replicon by the mean read depth across the chromosome.

### Confirming plasmid linearity

For the 12 linear-plasmid-positive isolates in our collection, reads from the day 1 (D1) ONT sequencing (see above) were aligned to their respective reference genomes using minimap2 v2.17 [19]. For each linear and circular plasmid sequence, we extracted all high-quality read alignments (read identity ≥80 %, alignment length ≥1000 bp) that aligned within 90 bp of the end of the plasmid reference sequence. For these reads, we calculated the proportion that extended ≥100 bp beyond the edge of the plasmid reference sequence (and, thus, were soft-clipped ≥100 bp by the read aligner). If the replicon from which the reads originated was linear, we would expect to see few or no such soft-clipped reads, because the 5′- and 3′-terminal ends of the plasmid ssDNA molecules should match the start and end of the reference sequence (see Fig. 1b). However, if the plasmid from which the reads originated was circular, we would expect to see many reads that are soft-clipped at the ends of the linearized reference sequence (see Fig. 1c).

### Linear plasmid characteristics and relationships

To compare gene content across plasmid sequences, all 86 linear plasmid sequences retrieved from *Enterobacteriaceae* genomes were annotated using Prokka v13.3 [20], and genes were clustered into homologous groups using panaroo v1.2.4 [21], with a threshold of 70 % amino acid identity to determine homology (details of the clusters can be found in Table S2). The panaroo gene presence/absence matrix (Table S3) was subjected to hierarchical clustering using *hclust* in R (with default settings, i.e. Euclidean distance and *ward.D2* clustering algorithm) to generate a dendrogram, which was cut into five phylogroups after visual inspection.

TIR length was calculated by taking each linear plasmid sequence, obtaining the reverse complement, and determining the length of sequence from the start of the forward and reverse complement sequences that were identical, with zero mismatches. Nearly all (except five) linear plasmid assemblies identified via nucleotide blast search of NCBI sequences had very small TIRs using this method (*n*=56, between 0 and 54 bp). We assume this is the result of artefacts in the assembly process, which we are unable to explore without the underlying sequence reads; therefore, plasmid sequences available only as publicly deposited assemblies without short reads were excluded from TIR length analyses. TIR sequences for the 25 linear plasmids generated from our assemblies were extracted, categorized into their respective phylogroups and aligned using the clustalo algorithm in SeaView [22] to identify regions of homology within phylogroups (Data S1). Nucleotide divergence between linear plasmid sequences was calculated by performing pairwise blastn alignments between all pairs of plasmids in the same phylogroup, and extracting the per cent identity of the longest hit.

### Detailed annotation of representative linear plasmids

To further explore gene function in these linear plasmids, we undertook detailed annotation for one representative per phylogroup (A, INF019; B, 1194/11; C, INF102; D, INF007; E, INF352). Each representative was annotated using the RASTtk pipeline [23–25]. We screened for Pfam domains for all genes identified by RAST with hmmscan [26] via the EMBL-EBI server using default parameters. Resulting Pfam domains for genes with hits are listed in Table S4. To determine whether any genes in the representative plasmids had homology to genes found in the *Enterobacteriaceae *, protein sequences were extracted from the RAST annotations and screened using blastp to the NCBI refseq_select database, restricting results to *Enterobacteriaceae *. Genes with at least 50 % protein identity to those in the *Enterobacteriaceae* were considered sufficiently similar to have a similar function.

To explore the toxin–antitoxin systems in more detail, we screened these sequences against databases of known systems using both TADB2 [27] and TASmania [28]. Hits are listed in Table S4. We reconstructed phylogenies of *relBE* and *vapBC* toxin–antitoxin systems using representative protein sequences from TADB2 (see Table S5 for a list of sequence accession numbers and details for those included). Protein sequences were aligned in SeaView using clustalo [22], and maximum-likelihood phylogenies were generated using iq-tree 2 [29] using the LG amnio acid model and performing 1000 ultrafast bootstrap replicates.

Representative plasmid annotations have been deposited in GenBank, accession numbers can be found in Table S1. To determine conservation of genes amongst plasmids in the same phylogroup, RAST annotations were matched with the Prokka annotations from the panaroo analysis.

### Trinucleotide profiles of linear plasmids and bacterial chromosomes

To investigate the potential donors of the linear plasmids into *Enterobacteriaceae *, we used *compseq* from the emboss package [30] to calculate the frequencies of all possible trinucleotides in each of our 12 *Klebsiella* linear plasmids, their host chromosomes, as well as one representative per bacterial species (*n*=47 893) as defined by the GTDB release 202 [31, 32]. We created a distance matrix using these frequencies with the *rdist* function in the R package *fields* (https://github.com/NCAR/Fields).

## Results and Discussion

### Identification of linear plasmids

We identified unusual structures in the assembly graphs of some *K. pneumoniae* in our in-house collection of genomes, which were consistent with linear plasmids with inverted repeats at either end (Fig. 1a, see Methods). We systematically screened for these structures in the assembly graphs of our in-house collection of *K. pneumoniae* species complex isolates, collected from human clinical infections or colonization [12, 13] in an Australian hospital (*n*=452), as well as a collection of *K. pneumoniae* isolates from Brazil (*n*=8) [14, 15], as described in Methods. This screen yielded 12 genomes harbouring linear plasmids (*n*=11, 2.4 % of genomes from Australia, and *n*=1, 12.5 % of genomes from Brazil); these include eight *K. pneumoniae* and four *Klebsiella variicola* (Table S1). The corresponding Australian isolates originated from nine patients, representing three instances of asymptomatic colonization (* K. pneumoniae* ST359, *K. variicola* ST386 and ST642), one instance of simultaneous gut colonization and pneumonia (* K. pneumoniae* ST37), and five instances of clinical infection (urinary tract infection with *K. pneumoniae* ST20, ST27, ST1449; wound infection with *K. pneumoniae* ST3073 and *K. variicola* ST347). The only extended-spectrum β-lactamase positive (which confers resistance to the third-generation cephalosporins) isolates amongst those with identified linear plasmids were two *K. variicola* ST347 isolated from the same patient 9 days apart.

The linear plasmids were a median of 33 775 bp in size (range 31 739–44 271 bp), including the TIRs at either end. To confirm our hypothesis that these plasmids were indeed linear molecules, rather than the typical circular plasmid structure, we undertook additional sequencing using long reads, and aligned the long reads to each linear plasmid (see Methods). Plasmids were considered linear if there were few soft-clipped bases from reads aligned at the start or end of the linear reference sequence (unlike a circular replicon, where many reads are expected to overlap the ends of the linearized reference sequence; see Fig. 1b). The 12 linear plasmids had a median of 3.5 % (range 0.6–32.4 %) soft-clipped start or end reads, compared to 98.5 % (range 92.3–100 %) for the circular plasmids (Figs 1b and S1, Table S1). Additionally, all but two linear plasmids (those from *K. variicola* ST347) were supported by reads (median *n*=70, range *n*=10 to 177) that spanned the full length of the plasmid, including both TIRs (Table S1). Importantly, the soft-clipped parts of the reads did not map to the other end of the plasmid sequence (as would be expected for a circular plasmid), rather, they were chimeric reads, where two unrelated DNA segments have fused during library preparation [33].

To investigate whether other linear plasmids are present in the *K. pneumoniae* species complex, we generated and screened assembly graphs for an additional 667 publicly available read sets, which represent a diverse set of (mostly human clinical) isolates from multiple continents including Africa, Asia and Europe (Table 1). Across this set of genomes, we identified linear plasmid graph structures in an additional 14 genomes (2.1 %, see Table 1). The corresponding isolates include 12 *K *. *pneumoniae* from humans (UK, Kenya, Cambodia, Brazil), one *K. pneumoniae* isolated from retail pork (USA) and one *Klebsiella africana* human blood isolate (Kenya).

Using as queries the sequences of the 25 linear plasmids that we identified from *Klebsiella* assembly graphs, we performed a blast search of NCBI sequences to identify homologues in other genomes (see Methods). It is possible that additional novel linear plasmids, with no homology to those we found, could exist in *Enterobacteriaceae *; however, to identify these would require assembling and inspecting the assembly graphs of all available read sets, which would require computational resources that are beyond our current capacity. However, our homology search revealed another 61 putative linear plasmid sequences; all were from *Enterobacteriaceae *, including *Klebsiella* (*n*=23, including 17 *K *. *pneumoniae *), *Salmonella enterica* (*n*=16, including pBSSB1), *Citrobacter* (*n*=8), *Enterobacter* (*n*=7), *Escherichia coli* (*n*=3), *Serratia marcescens* (*n*=2), *Phytobacter diazotrophicus* (*n*=1) and *Kluyvera ascorbata_B* (*n*=1) (Table S1). Genomes harbouring linear plasmids came from a wide variety of sources, including bacteria isolated from water (*n*=19), humans (*n*=13), food (*n*=4), animals (*n*=3) and plants (*n*=1) (Table S1). Amongst the linear-plasmid-positive *K. pneumoniae* were well-known carbapenemase-producing and extended-spectrum β-lactamase producing clones: ST340 (*n*=3, KPC-4 and CTX-M-15), ST258 (KPC-2 and SHV-12), ST11 (*n*=1, KPC-2 and SHV-12), ST147 (*n*=1, OXA-181 and CTX-M-15). Hundreds of genomes of each of these clones are present in the NCBI database and the vast majority do not harbour linear plasmid sequences, suggesting that the linear-plasmid-positive variants are rare, and likely result from recent horizontal transfer but this has not resulted in clonal expansion during which the plasmid has been stably maintained. This is in contrast to the recent report in *Enterococcus faecium* where the linear plasmid *pELF_USZ* was stably maintained in a host lineage during >2 years of clonal spread in a hospital [4].

### Characteristics of linear plasmids in *Enterobacteriaceae*

**Fig. 2.**
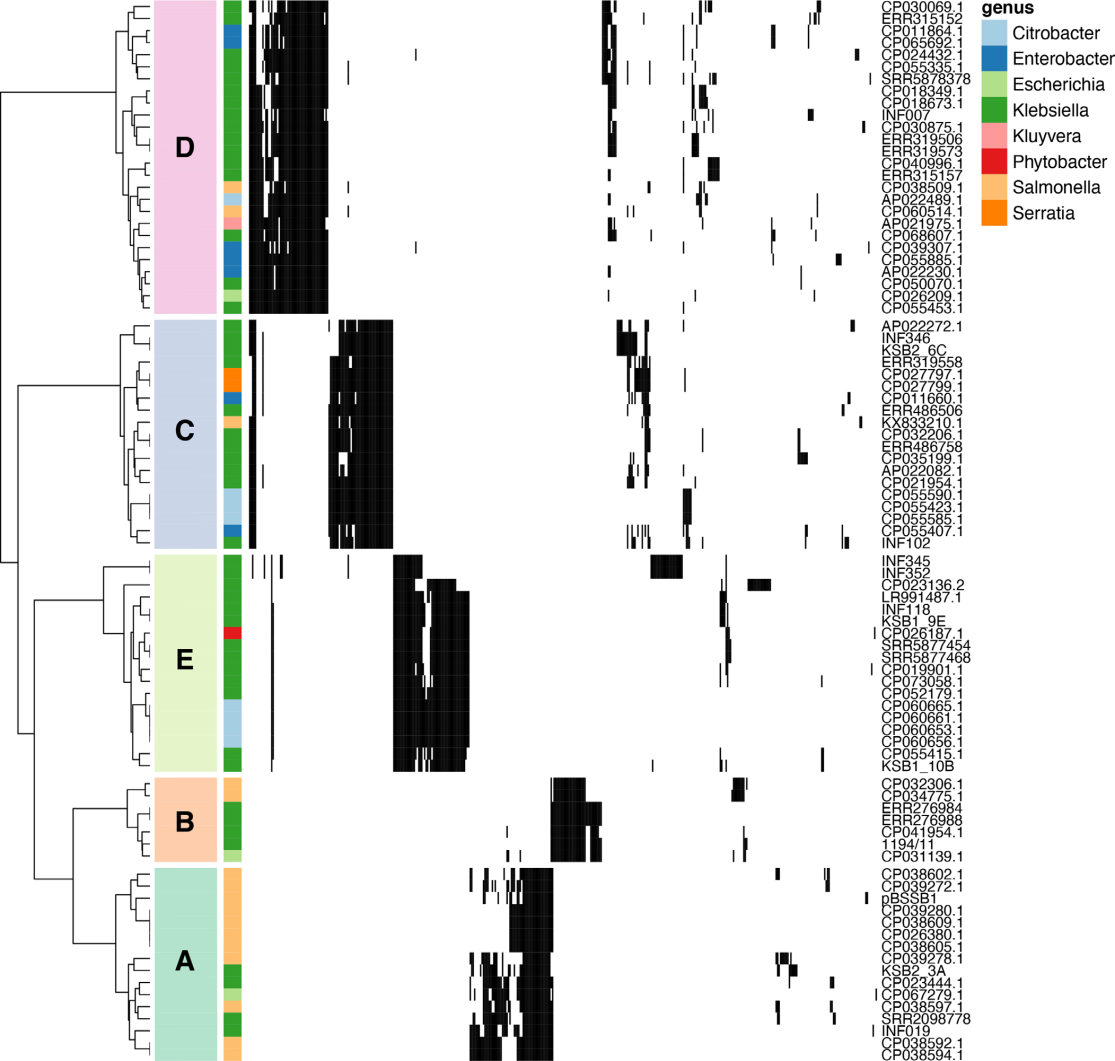
Hierarchical clustering of linear plasmids based on gene content. Plasmids were clustered with the *hclust* algorithm using the *ward.D2* method, and divided into five phylogroups (labelled in coloured boxes). Rows are annotated with the bacterial genus each linear plasmid was found in, as per the key. Black indicates the presence of a gene, white the absence. Plasmids are labelled with their names as per Table S1, and details of each gene can be found in Table S2.

**Fig. 3.**
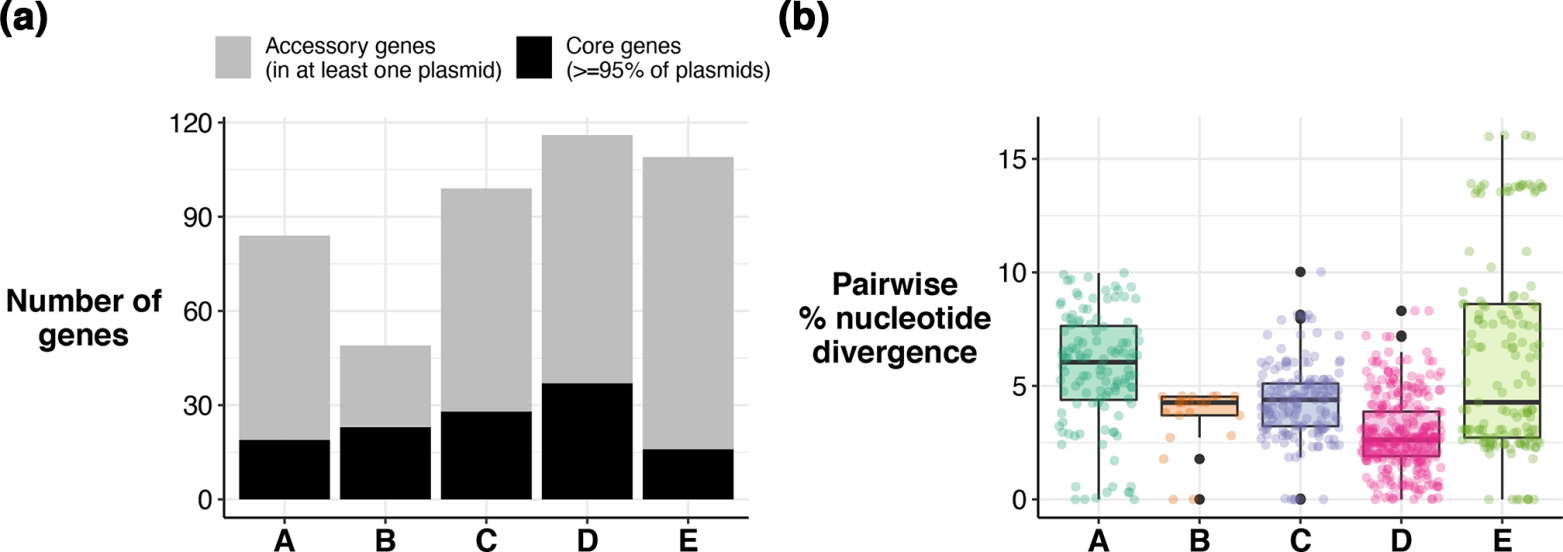
Core and accessory gene content by phylogroup, and nucleotide divergence by phylogroup. (a) Number of core and accessory genes in each phylogroup. Bar height indicates the total number of genes found in at least one linear plasmid in each phylogroup. Black indicates the number of core genes (found in ≥95 % of plasmids); grey indicates the number of accessory genes. (**b)** Distribution of pairwise nucleotide divergence within each phylogroup. Boxplots show the median (thick black line), first and third quartiles (edges of box), and solid lines give the 1.5× the interquartile range. Outliers are shown as black dots. Individual values are shown as coloured dots.

**Fig. 4.**
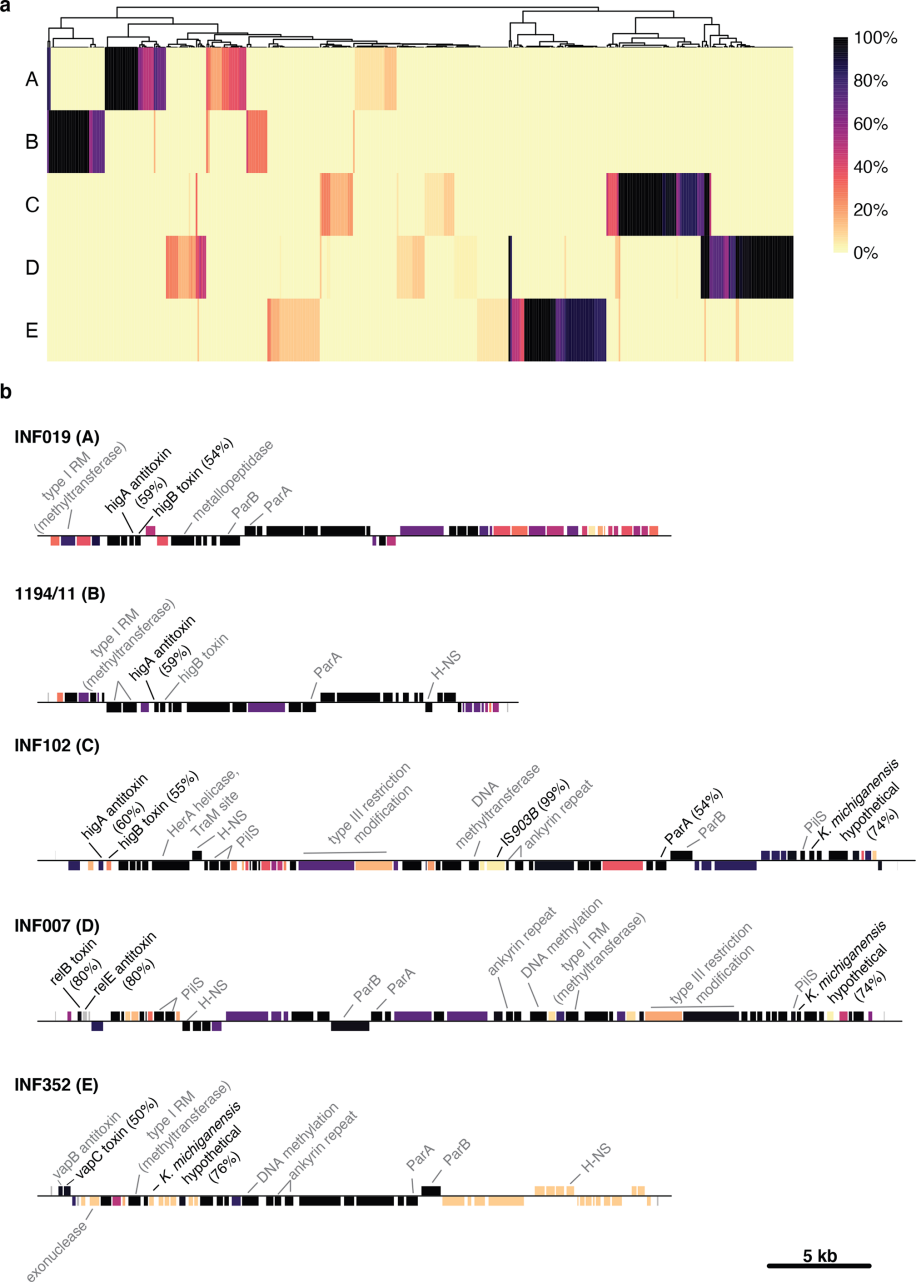
Conservation and function of genes in each phylogroup. (a) Heatmap showing proportion of plasmids with each gene by phylogroup. Columns are genes, clustered using *hclust*; rows are phylogroups (unclustered). Colour within each cell indicates the proportion of plasmids carrying each gene, as per the key. (**b)** Gene maps of one representative plasmid per phylogroup. Genes are indicated by blocks (above the line, forward orientation; below the line, reverse orientation) and coloured by conservation in their phylogroup. Genes with ≥50 % homology to known genes in *Enterobacteriaceae* are indicated by black lines and text, with gene homology shown in brackets. Genes with detected Pfam domains are indicated by grey lines and text. Details of each gene can be found in Table S4.

We compiled the full set of 86 linear plasmid sequences (25 identified from assembly graphs, plus 61 inferred from homology via blast) and clustered them by their gene content (see Methods). This revealed five distinct linear plasmid phylogroups (which we labelled A–E, see Fig. 2, Tables S2 and S3), with very little gene sharing between phylogroups (genes defined as homologous if they had >70 % nucleotide identity). Each phylogroup included sequences from multiple genera, notably all five phylogroups were detected in both *Klebsiella* and *Salmonella* (Fig. 2). No genes were present across more than two phylogroups, but each phylogroup had a core set of genes found in ≥95 % of plasmids in that group; these represented between 15 % (phylogroup E) to 47 % (phylogroup B) of all genes found in that phylogroup (Figs 3a and 4a). Nucleotide diversity within phylogroups varied (Fig. 3b), with phylogroup A displaying significantly greater pairwise divergence across the full plasmid sequence than phylogroups B, C and D (mean 6 % divergence vs mean 2.6–4.2 %, *P* <1×10^−16^ using Wilcoxon test for A vs B, C or D). Phylogroup E showed a high range in divergence (0–16 %, mean 4.2 %), due to the presence of two divergent subgroups (see Fig. 2).

The vast majority of genes annotated in each linear plasmid were hypothetical proteins and had no close homologues in other *Enterobacteriaceae* genomes (Fig. 4b). However, there were a few reference plasmid genes (*n*=55, 20%) for which we were able to obtain some form of functional annotation based on protein sequence homology or protein domain matches (see Methods, Table S4). Most of these annotations were for genes encoding proteins likely relevant to basic plasmid maintenance functions. All five phylogroups carried genes with type II toxin–antitoxin domains (see Table S4), which are often found on plasmids and can enable plasmid maintenance by performing post-segregational killing of daughter cells that do not carry the plasmid [34]. These systems were core in all phylogroups. Phylogroups A, B, C and D each carried a *relBE* family system (65–83 % pairwise homology between variants in phylogroups B, C and D; A carried a distinct variant), whilst phylogroup E carried a *vapBC* system (Fig. 4b). These toxin–antitoxin clusters generally had at least one gene of the pair encoding a protein with ≥50 % protein homology to toxin–antitoxin systems found in *Enterobacteriaceae* (Fig. 4b, Table S4). Comparison of the sequences found in our linear plasmids with known sequences in the toxin–antitoxin database TADB2 [27] demonstrated that most of these sequences were divergent from currently described toxin–antitoxin systems, with ≤53 % amino acid identity (Figs 4b and S2, Table S4). The *relBE* systems from phylogroups A, B and C clustered together, with the nearest relative belonging to *Bordetella* (42 % amino acid identity) (Fig. S2a, b). The system in *Bordetella* belongs to *higBA*, a subsystem within the *relBE* family where the toxin is encoded upstream of the antitoxin – this arrangement was also present in phylogroups A–C, suggesting these too are *higBA* systems (Fig. 4b). Phylogroup D also carried a *relBE* system that had 88 % protein homology with sequences found in *Enterobacteriaceae *, and clustered most closely with *S. enterica* (80 % amino acid identity) (Fig. S2a, b). In phylogroup D, the toxin was encoded downstream of the antitoxin, indicating it is not a *higBA* system (Fig. 4b). The *vapBC* system in phylogroup E had no close relatives, sharing most homology with systems in *Neisseria* (37 % amino acid identity) (Fig. S2c, d).

Pairs of adjacent genes encoding novel proteins with Pfam matches to the partitioning proteins ParA (PF13614 or PF01656) and ParB (PF18821) were detected as core in each phylogroup (Fig. 4, Table S4). These likely contribute to the control of plasmid segregation into daughter cells [35]; however, the ParA sequences shared only 25–46% amino acid identity with homologues detected in other *Enterobacteriaceae *, and the ParB sequences had no homologues detected in other *Enterobacteriaceae* (see Table S4). We were unable to detect any genes with homology or Pfam domains to known replication systems. Sequences with homology to the transcriptional dual regulator *hns* were identified in all phylogroups except A (Fig. 4b); however, the encoded proteins were highly divergent from one another (27–66 % pairwise homology between phylogroups) and the genes were classed as separate gene groups by panaroo (Table S2). Hns is a global regulator that can be plasmid encoded [36], and can regulate expression of both plasmid- and chromosomally encoded genes [37], impacting a wide range of phenotypes, including virulence [38], expression of genes on foreign DNA [39] and growth conditions [40]. Proteins with hits to known restriction/modification domains were also identified in all reference plasmids, these are frequently encoded by mobile elements and can function as toxin–antitoxin systems to force maintenance of those elements. Phylogroup A was the only phylogroup in which flagellin genes were identified, in *n*=7/16 plasmid sequences. One of these was plasmid pBSSB1, and the other six were all linear plasmids from *Salmonella enterica* serovar Senftenberg isolated from Switzerland [11]. Phylogroups C and D both carried three core genes apiece harbouring PilS (type IV pilin) domains (Fig. 4b), which could potentially function as adhesins.

**Fig. 5.**
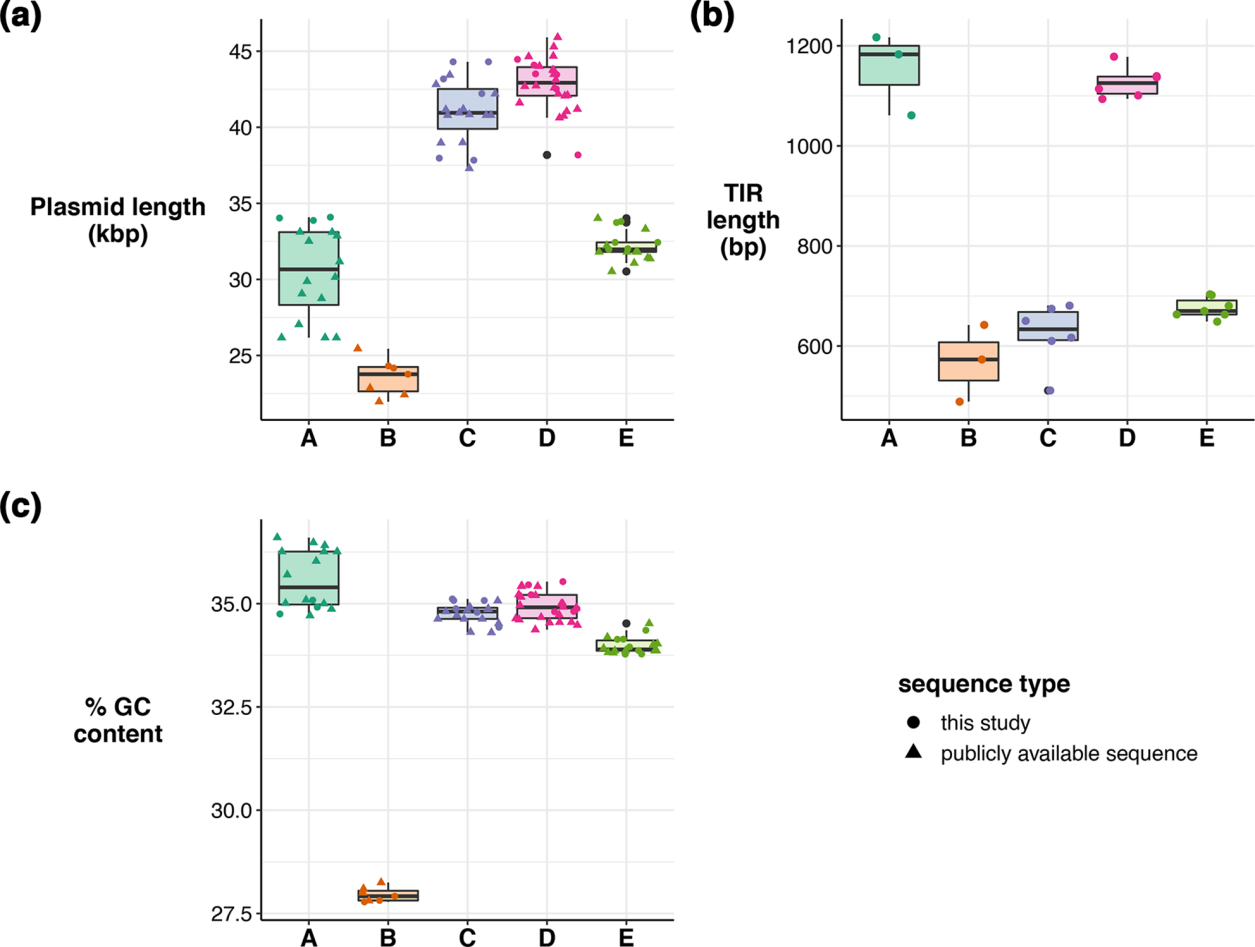
Characteristics of linear plasmid phylogroups. (a) Distribution of plasmid lengths in kbp. Boxplots show the median (black line), first and third quartiles (edges of box), and solid lines give the 1.5× interquartile range. Outliers are shown as black dots. Individual values are shown as coloured dots or triangles, with the shape indicating the origin of the sequence, as per the key. (b) Distribution of TIR lengths in bp, as per (a). Publicly available sequences are not represented in this plot due to assembly errors in the TIR region. (c) Distribution of G+C content, as per (a).

All five phylogroups differed substantially from one another in their basic characteristics, including plasmid length, TIR length and G+C content. Phylogroups D and C had the longest plasmids (medians 40.9 and 42.9 kbp, respectively), and phylogroup B the smallest (median 23.7 kbp; Fig. 5a). We calculated the size of TIRs by aligning the beginning of each plasmid to the reverse complement of itself (see Methods). We were able to detect a TIR in 57 of the linear plasmid sequences. Those without a TIR (*n*=29) were all identified in publicly available assemblies that were assembled using a variety of methods, and we hypothesize that the lack of TIR sequence is most likely due to incomplete or fragmented assembly of the plasmid, rather than lack of TIR in the sequenced molecules. For plasmids where we performed the assembly in-house, we found that the length of the TIR differed substantially between phylogroups, with phylogroups A and D having the longest TIRs (medians 1168 and 1074 bp, respectively), whilst phylogroups B, C and E had TIRs of approximately half that length (medians 542, 530 and 670 bp, respectively; Fig. 5b). There was a high level of sequence conservation for TIRs within phylogroups, with a median of 89–97 % similarity in this region in phylogroups A–D (Data S1, Fig. S3). Phylogroup E was more diverse with an overall similarity of 65 %; however, inspection of the alignments revealed that this phylogroup carried two distinct TIR sequences, with a median of 89–99 % TIR sequence identity within each TIR grouping (Data S1, Fig. S3). Finally, mol% G+C for the linear plasmids was very low in comparison to the normal chromosomal mol% G+C range for *Enterobacteriaceae *, which is typically ~50 mol% (median 57 mol% for the *Klebsiella* carrying linear plasmids). All linear plasmid phylogroups had G+C <40 mol%, with phylogroup B having the lowest out of all the phylogroups (median 28 mol%, compared to 34–35 mol % for other phylogroups, *P*<2.5×10^−4^ for all comparisons, Wilcoxon test; Fig. 5c).

### Potential donors of linear plasmids and their stability in *Klebsiella*

Given that linear plasmids are rare in *Klebsiella* and have a significantly lower mol% G+C than their host chromosomes, we assume that *Enterobacteriaceae* are unlikely to be the typical hosts for these plasmids. We used trinucleotide frequencies as a genomic signature to attempt to identify potential original hosts of these plasmids by calculating the distance between our linear plasmids, their *Klebsiella* host chromosomes, and one representative per bacterial species defined in the GTDB (see Methods). The 12 *Klebsiella* linear plasmids clustered separately from their corresponding host chromosomes, with a mean distance of 2.3 between the chromosomes and linear plasmids (Fig. S4). The *Klebsiella* chromosomes were much more similar to each other than the linear plasmids (mean pairwise distance of 0.07 between chromosomal sequences vs 1.27 between pairs of linear plasmid sequences), and clustered closely with other representatives of *Klebsiella* in the GTDB (nearest neighbour accession no. GCF_000742135.1, distance 0.09). The linear plasmid from 1194/11 clustered most closely to the firmicute DUOC01 sp012839065 (accession no. GCA_012839065.1, distance 1.3). This organism belongs to a strain from the class *Thermosediminibacteria*, which was detected in a metagenomic sample obtained from an anaerobic digester [41]. The other 11 linear plasmids were their own nearest neighbours (median pairwise distance 1.09), the closest GTDB profile was *Proteobacteria* isolate *Neptuniibacter* sp002435145 (accession no. GCA_002435145.1, mean distance 1.15 to the 11 plasmids). This organism belongs to the order *Pseudomonadales *, and was detected in a marine environment [42].

**Fig. 6.**
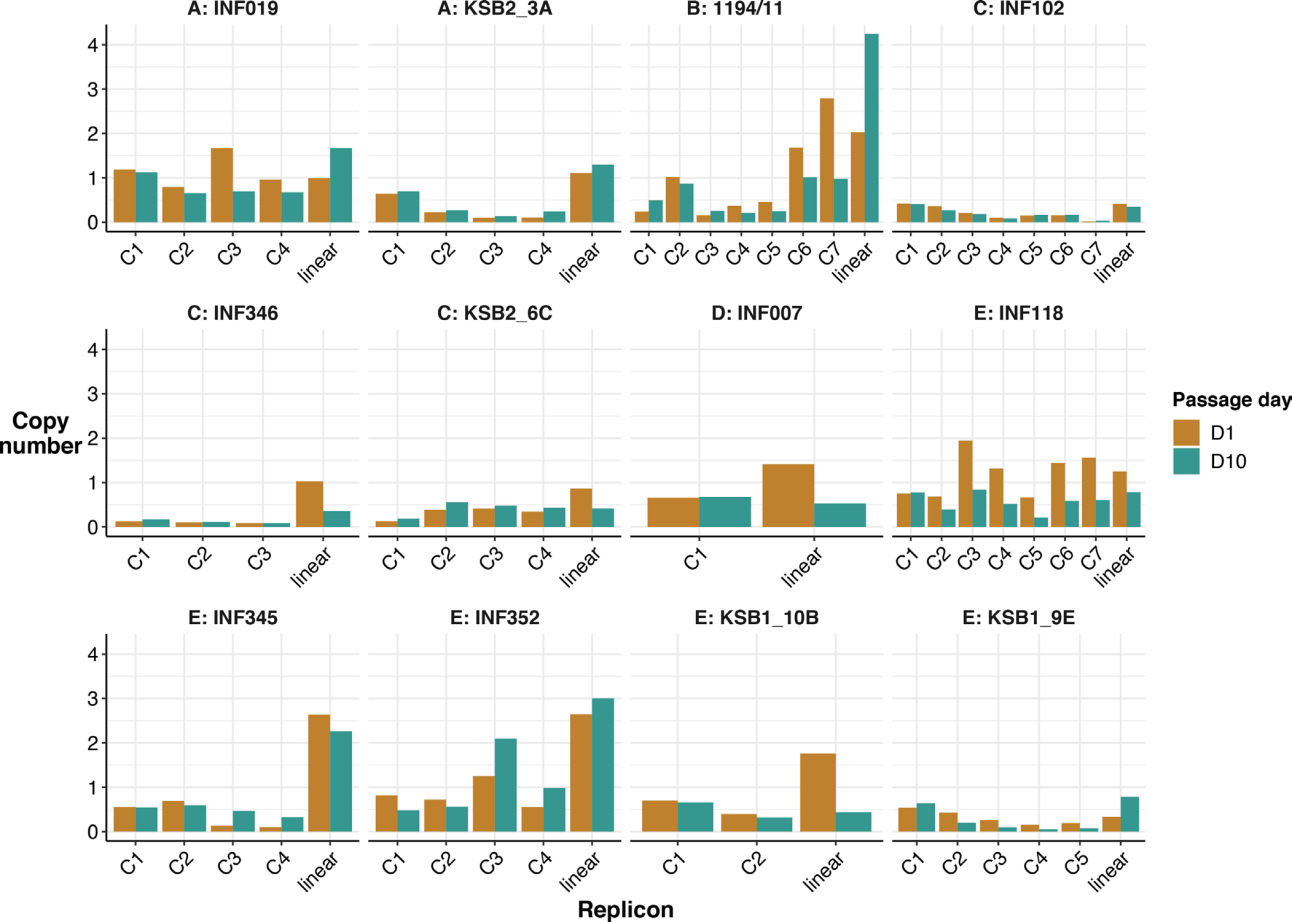
Estimated copy number of all plasmid replicons in each genome. The height of each bar indicates copy number, bars are coloured by passage day, as per the key. Each pair of bars represents a plasmid: C[N] indicates a circular plasmid, linear indicates the linear plasmid in that genome.

To understand whether linear plasmids could be stably maintained within *Klebsiella *, we undertook passage experiments on the 11 *Klebsiella* genomes carrying linear plasmids in our collection. We performed long-read sequencing on all parental isolates (D1), passaged each isolate 10 times (one passage per 24 h period), and then performed long-read sequencing on the final D10 isolates (see Methods). We found that all plasmids, both linear and circular, were maintained in all genomes across 10 passages (Fig. 6). Linear plasmid copy number was generally estimated at ~1 per cell at both D1 and D10, with the exception of 1194/11 (the only representative of phylogroup B), which had a copy number of 2–4, and two of the phylogroup E plasmids (strains INF345, INF352) with copy number ~2 (see Fig. 6).

### Conclusions

Here, we provide the first (to our knowledge) collection of linear plasmids in the *K. pneumoniae* species complex alongside a detailed description of their characteristics. Our data show these plasmids are uncommon in *Klebsiella* and other *Enterobacteriaceae* species, but can be stably maintained in distinct *K. pneumoniae* strains, and are occasionally detected in representatives of the globally distributed multidrug-resistant clones and other diverse *Enterobacteriaceae* [5] consistent with horizontal transfer. These linear plasmids appear to be distinct to those previously described in other bacterial species like *Streptomyces* and *Borrelia *, as their gene content is distinct and their trinucleotide frequencies do not indicate these species as the likely original hosts. The novel *Klebsiella* linear plasmids described here do not carry any known antimicrobial resistance, virulence or metabolic genes; however, carriage of a linear plasmid has previously been shown to provide a metabolic advantage for vancomycin-resistant *Enterococcus faecium* in the human gut [4] and to enable flagellar antigen switching in *Salmonella enterica* Typhi. By making freely available these linear plasmid sequences and representative isolates that carry them, we hope to facilitate future research into the function and potential evolutionary or clinical significance of these enigmatic replicons.

## Supplementary Data

Supplementary material 1

Supplementary material 2
