# Supplementary material for: Linear plasmids in Klebsiella and other Enterobacteriaceae
Source: Microb Genom. 2022 Apr 13;8(4):000807. doi: 10.1099/mgen.0.000807 (PMC9453081; doi:10.1099/mgen.0.000807)
Supplement: Supplementary material 1 [file mgen-8-0807-s001.pdf]

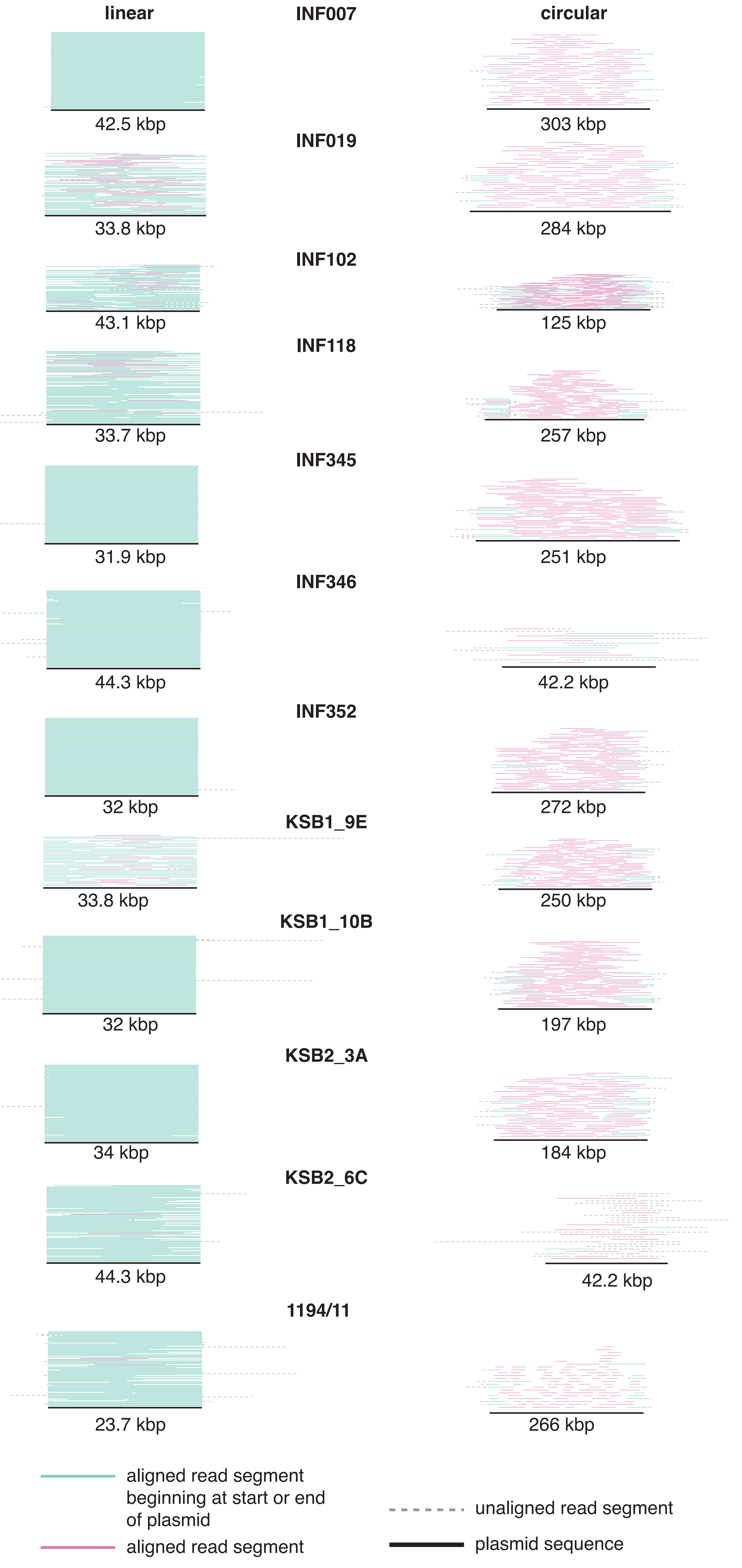

Figure S1: Long read alignments to linear plasmids and one representative circular plasmid per Klebsiella genome. The total number of alignments shown is capped at 100 to improve visualisation. The plasmid sequence is the thick black line at the bottom, and reads aligning to the plasmid are shown in green if the alignment starts at the beginning or end of the plasmid sequence, or pink if the alignment starts elsewhere. Segments coloured dotted grey indicate regions of the read that do not align.

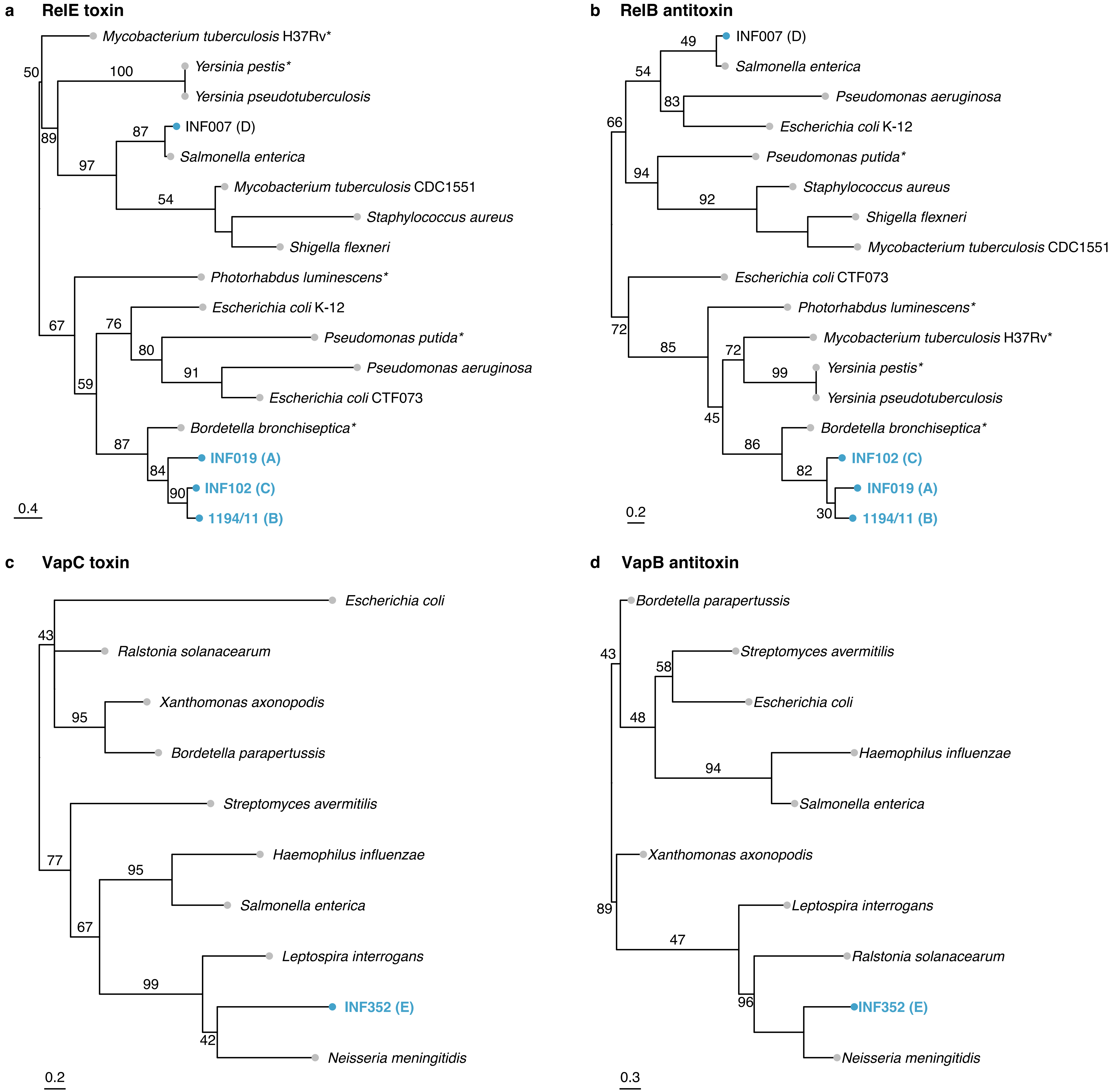

Figure S2: Maximum-likelihood phylogenies of type II toxin-antitoxin systems in various bacterial taxa. a, Phylogeny of RelE toxins from various bacterial species as well as toxin genes found in the representative linear plasmids. Tree is midpoint rooted, with bootstrap values given on each internal node. Scale bar shows number of substitutions per site. Grey, reference sequences; blue, linear plasmid sequences. Species with asterisks indicate that these sequences come from the HigBA subfamily. Phylogroups for each linear plasmid are shown in brackets. b, Phylogeny of RelB antitoxins. Species with asterisks indicate that these sequences come from the HigBA subfamily, other details as per a; c, Phylogeny of VapC toxins, other details as per a; d, Phylogeny of VapB antitoxins, other details as per a.

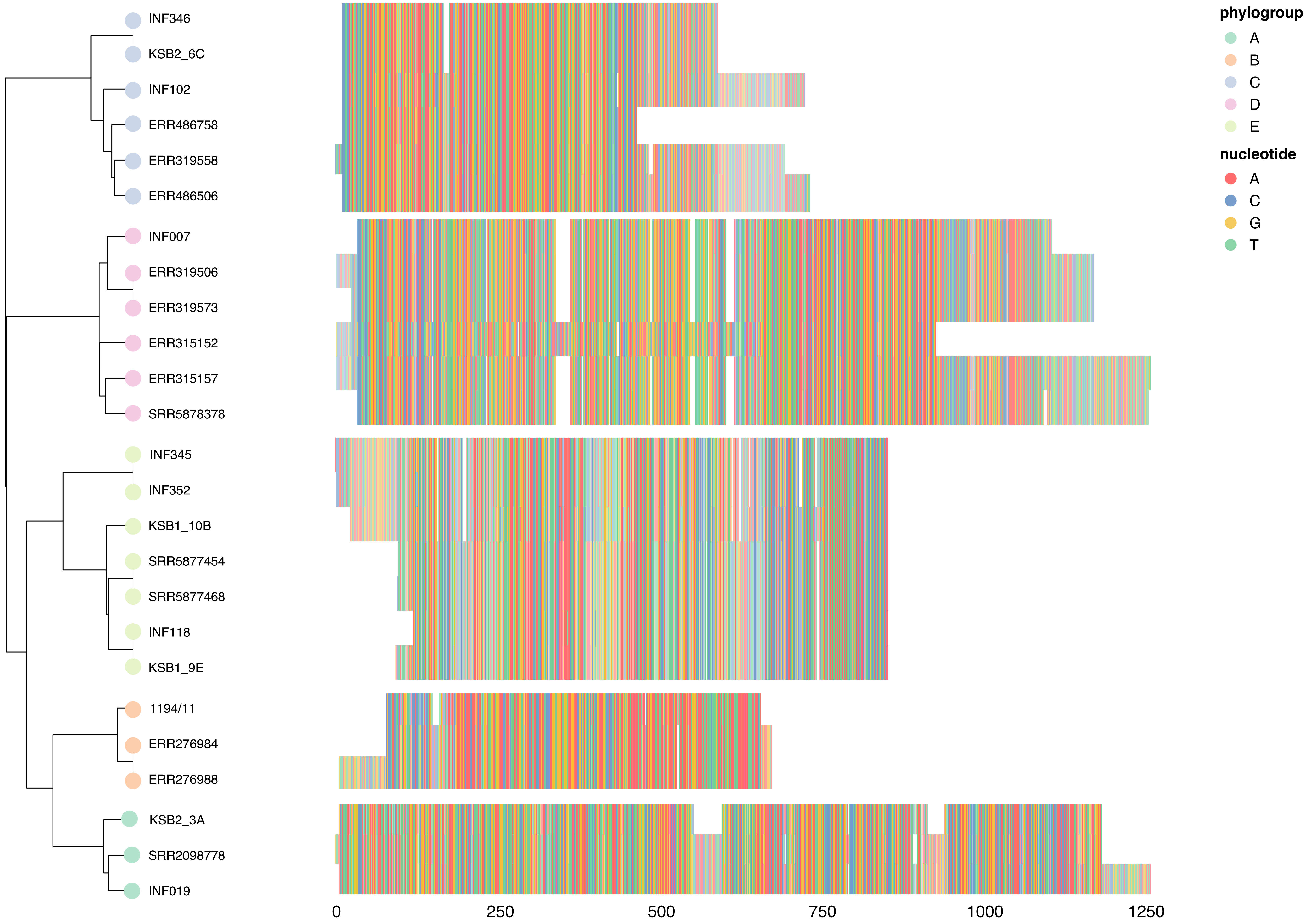

Figure S3: TIR sequence alignments within each phylogroup. Linear plasmid sequences are clustered by gene content, with the phylogroup indicated by tip colour and coloured as per legend. TIR sequences are aligned within each phylogroup, where each colour represents a different nucleotide as per legend. Colour intensity indicates level of conservation at that position (pale=low; intense=high).

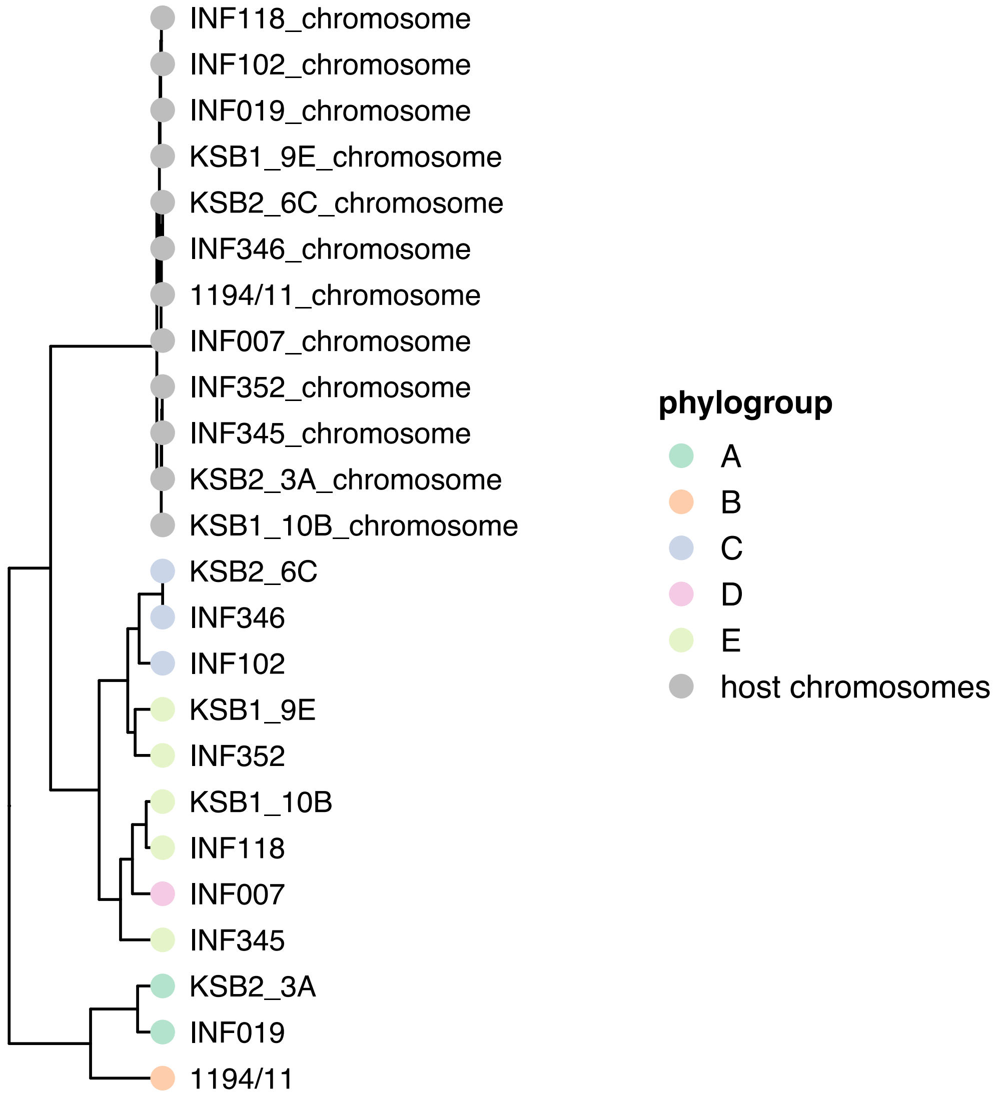

Figure S4: Cluster dendrogram of trinucleotide frequencies for the 12 linear plasmids and their host Klebsiella chromosomes. Trinucleotide frequencies were clustered using hclust. Tips are coloured by phylogroup or chromosome (as per legend).
